# Supplementary material for: Trophic niche but not abundance of Collembola and Oribatida changes with drought and farming system
Source: PeerJ. 2022 Jan 13;10:e12777. doi: 10.7717/peerj.12777 (PMC8761369; doi:10.7717/peerj.12777)
Supplement: Supplemental Information 1 [file peerj-10-12777-s001.pdf]

Table S1: Soil characteristics in the conventional (“ConMin” of the DOK-trial) and the organic (“BioDyn”) farming system. Mean ± SE.

	Farming system	
	conventional	organic
pH (in water)	6.03 ± 0.1	6.63 ± 0.04
WHC (0-10 cm) [%]	39.16 ± 0.89	42.03 ± 1.23
bulk density (0-10 cm) [g/cm³]	1.21 ± 0.02	1.17 ± 0.03
C_{tot} [%]	1.27 ± 0.05	1.60 ± 0.03
N_{tot} [%]	0.13 ± 0	0.17 ± 0
PO4-P [µg/g DW]	0.00104 ± 0.00005	0.001 ± 0.00005
P [mg/g DW]	0.827 ± 0.014	0.801 ± 0.024
