# Supplementary figures and images for: Trophic niche but not abundance of Collembola and Oribatida changes with drought and farming system
Source: PeerJ. 2022 Jan 13;10:e12777. doi: 10.7717/peerj.12777 (PMC8761369; doi:10.7717/peerj.12777)

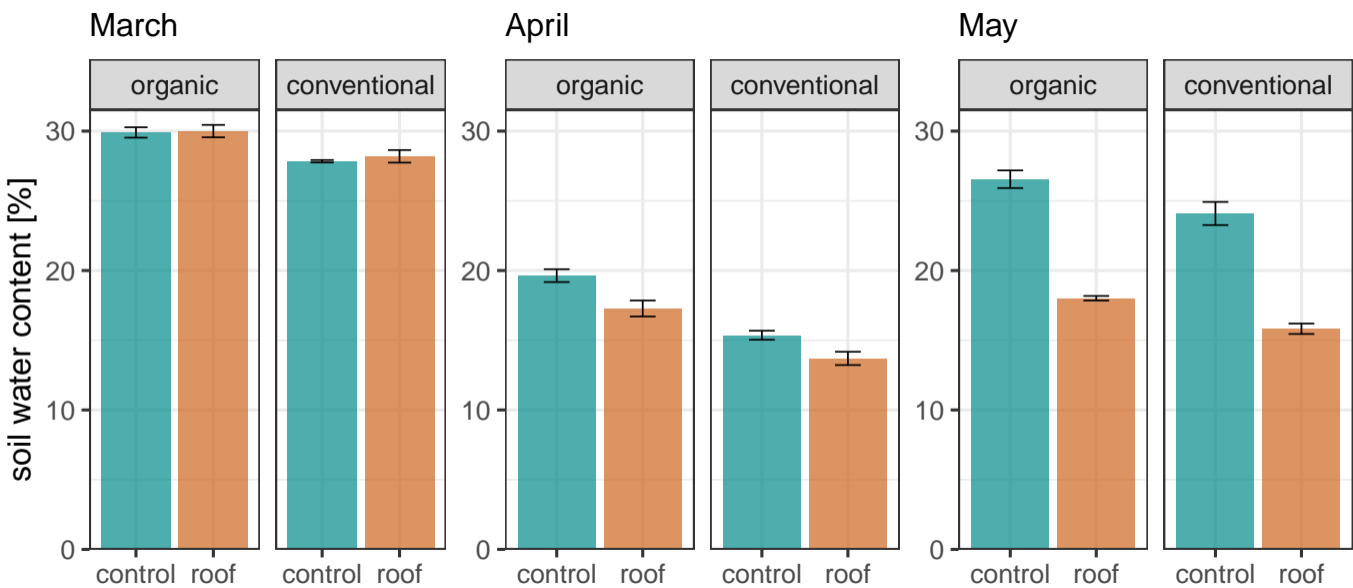

Supplement: Supplemental Information 2 — Gravimetric soil water content (0–20 cm depth) in control and drought treatments (roof) in wheat fields under organic and conventional management in March (before roof establishment), April and May (sampling of soil animals); means ± SE based on four replicates; modified from (Meyer et al., 2021). [file peerj-10-12777-s002.pdf]

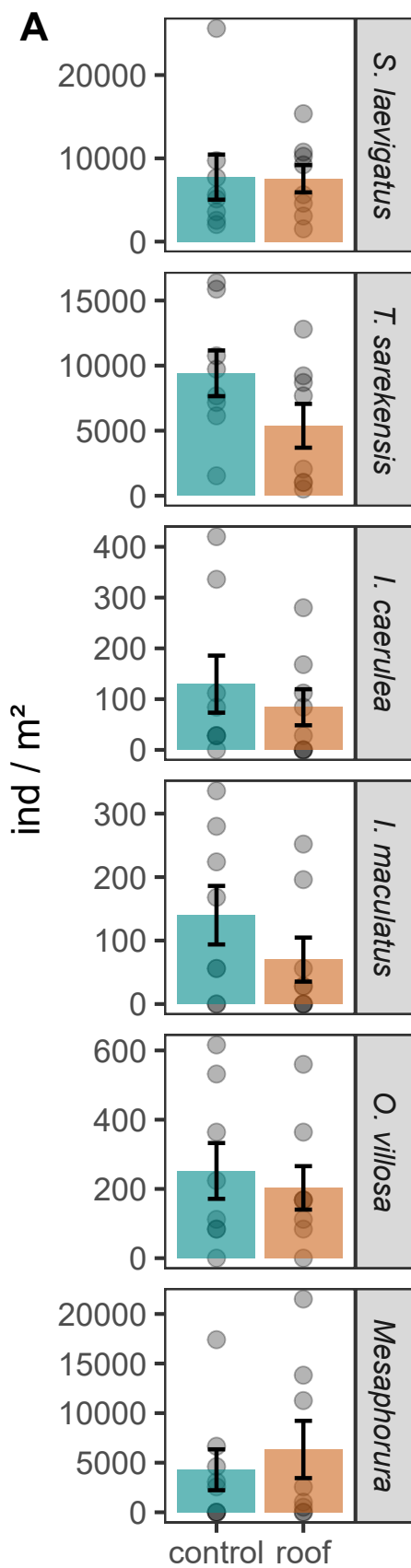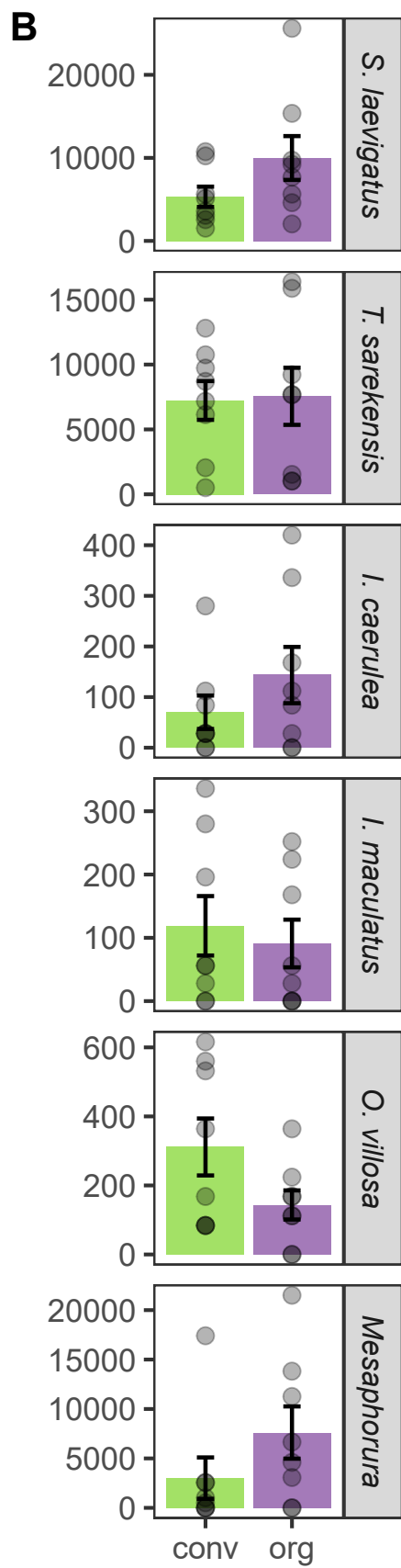

Supplement: Supplemental Information 3 — Abundance (mean ± standard error) of two oribatid mite (Scheloribates laevigatus, Tectocepheus sarakensis) and four Collembola species (Isotoma caerulea, Isotomurus maculatus, Orchesella villosa, Mesaphorura sp.) in (A) control and drought treatments (roof) and (B) under conventional (conv) and organic (org) management. [file peerj-10-12777-s003.pdf]

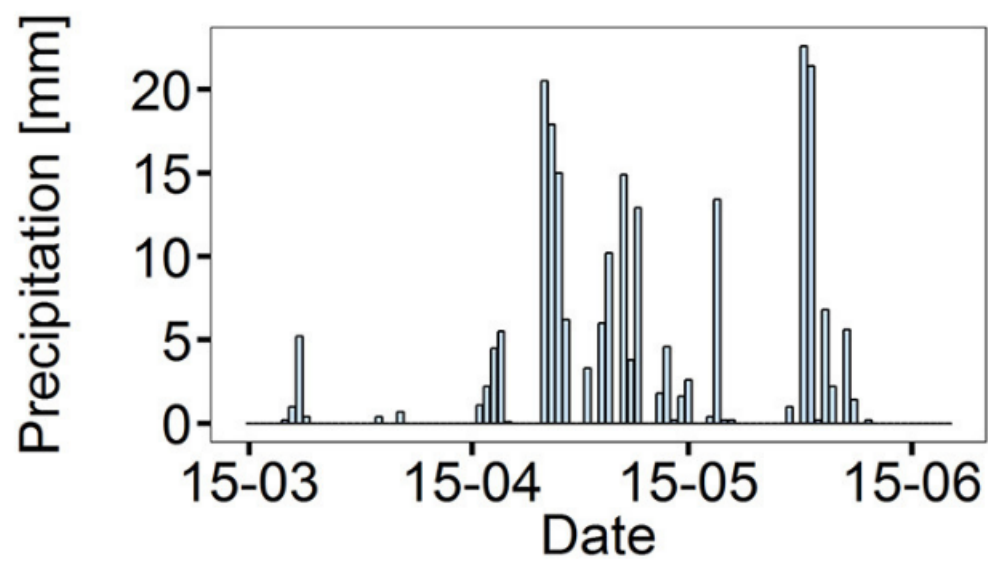

Supplement: Supplemental Information 4 — Data between April 5 to May 8, 2017 derived from the online database http://www.bodenmessnetz.ch (station in Therwil), all other data was recorded by the on-site weather station (Campbell-CR1000); modified from Kundel et al. (2018). [file peerj-10-12777-s004.pdf]
